# *Salmonella* Typhi Stool Shedding by Patients With Enteric Fever and Asymptomatic Chronic Carriers in an Endemic Urban Setting

**DOI:** 10.1093/infdis/jiab476

**Published:** 2021-09-29

**Authors:** Farhana Khanam, Thomas C Darton, James E Meiring, Protup Kumer Sarker, Prasanta Kumar Biswas, Md Amirul Islam Bhuiyan, Nazmul Hasan Rajib, Susan Tonks, Andrew J Pollard, John D Clemens, Firdausi Qadri

**Affiliations:** 1 icddr,b (International Centre for Diarrhoeal Disease Research, Bangladesh), Dhaka, Bangladesh; 2 School of Medical Science, Griffith University, Gold Coast, Australia; 3 Department of Infection, Immunity and Cardiovascular Disease, and the Florey Institute for Host-Pathogen Interactions, University of Sheffield, Sheffield, United Kingdom; 4 Oxford Vaccine Group, Department of Paediatrics, University of Oxford, and the NIHR Oxford Biomedical Research Centre, Oxford, United Kingdom

**Keywords:** Typhoid fever, stool shedding, chronic carriers, transmission of *S.* Typhi

## Abstract

The burden of *Salmonella enterica* serotype Typhi (*S.* Typhi) shedding in stool and its contribution to transmission in endemic settings is unknown. During passive surveillance *S*. Typhi shedding was seen during convalescence in 332 bacteremic patient with typhoid, although none persisted at 1-year follow-up. Anti–virulence capsule (Vi)–immunoglobulin (Ig) G titers were measured in age-stratified cohort of serosurveillance participants. Systematic stool sampling of 303 participants with high anti–Vi-IgG titers identified 1 asymptomatic carrier with shedding. These findings suggest that ongoing *S.* Typhi transmission in this setting is more likely to occur from acute convalescent cases, although better approaches are needed to identify true chronic carriers in the community to enable typhoid elimination.

Humans are the only natural host and reservoir of *Salmonella enterica* serotype Typhi (*S*. Typhi), the causative organism of typhoid fever. The multiplication of *S*. Typhi outside the human host is not robust, although some ability to survive in the environment for an extended time is theoretically required for transmission [1]. Typhoid is transmitted by consumption of food and water contaminated by bacteria shed during acute disease or by chronic carriers [2]. After recovery from acute disease, the infections fail to clear in approximately 2%–5% of patients [3, 4]. To maintain this carriage state, *S*. Typhi colonizes the gallbladder to form dense bacterial biofilms on gallstones and other surfaces from where organisms are shed intermittently and are excreted in stool [5].

While implementing preventive measures, such as vaccination, will lead to elimination of typhoid from endemic regions, additional interventions are needed to stop the ongoing transmission of *S*. Typhi from chronic carriers. This requires tools to accurately identify and appropriately treat individuals shedding *S.* Typhi. Detection of chronic carriers is challenging, however, as the majority of carriers are asymptomatic and up to 25% do not recall symptoms or an episode compatible with acute typhoid fever [4]. Microbiological culture of bile, gallstones, or gallbladder tissue from individuals undergoing cholecystectomy are reference-standard approaches to the accurate, but often coincidental, diagnosis of chronic carriage. Methods to obtain this material (surgical or string test) are not feasible at large (community or population) scales. Culture of serially collected stool specimens is not easy to perform and may only have a limited increase in diagnostic yield [6]. Some studies have demonstrated that high immunoglobulin (Ig) G isotype antibody titers to the virulence capsule (Vi) of *S.* Typhi (anti–Vi-IgG) can allow the identification of asymptomatic carriers as the apparent source of infection in outbreak settings, although this approach has not been evaluated prospectively at the population level [5].

The rate of stool shedding and contribution to transmission from short-term, convalescent shedding and longer-term, temporary or chronic carriage is unknown in endemic settings. Bangladesh is an area of high endemicity for typhoid fever and therefore a well-placed setting to explore these unknowns. A comprehensive population-based study was carried out in a densely populated area of Dhaka, Bangladesh, under the umbrella of the Strategic Typhoid Alliance Across Africa and Asia (STRATAA), to detect the occurrence and duration of *S*. Typhi shedding from both acutely infected patients and asymptomatic chronic carriers detected by serosurveillance.

## METHODS

### Enumeration of Population and Passive Surveillance

Passive surveillance for acute typhoid fever was performed in wards 3 and 5 of the Mirpur area in Dhaka city, Bangladesh [7]. A baseline census and 6-monthly updates were carried out to enumerate the population and to collect baseline characteristics.

In passive surveillance, patients presenting with a history of fever for ≥72 hours or axillary temperature ≥38.5ºC were enrolled. In a subsequent amendment to the study protocol, the inclusion criteria were changed to include fever ≥48 hours or axillary temperature ≥38.0ºC. At enrollment (day 0), blood and stool specimens were collected for culture to confirm acute typhoid cases. Study participants with typhoid diagnosed by blood culture and whose households were enrolled in the census survey were identified as index patients. Index patients were treated with antibiotics determined by bacterial susceptibility. Stool specimens were collected on days 30 and 180 to identify prolonged shedding. In those found to be shedding at these time points, another stool specimen was collected 1 year after study enrollment. The participants who were blood culture negative but stool culture positive underwent the same schedule of follow-up and household contact screening. They were also identified as index patients if their households were enrolled in the census.

Immediately after confirming the index cases, all household members were invited to participate in the study and stool specimens were collected on days 0 and 30 from the household contacts who have provided consent. Another stool specimen was collected on day 180 when culture results were positive at day 0 and/or day 30. Depending on the culture result at day 180, a follow-up stool specimen was recommended at 1 year to confirm continued shedding.

### Serosurvey

Apparently healthy participants, stratified by age (0–4, 5–9, 10–14, or >14 years) were randomly selected from the census population and enrolled in a serosurveillance component. Recruitment of selected participants and collection of specimens was carried out throughout the year, in 3-monthly blocks. Blood specimens were collected from the participants on days 0 and 90 after enrollment, and anti–Vi-IgG responses were measured. A Vi-IgG threshold value was calculated using the first 1000 specimens collected, based on the 97th centile of titers measured in participants <15 years or the 95th centile of participants ≥15 years of age. This provided anti–Vi-IgG thresholds of 96.2 and 57.1 ELISA Unit/mL, respectively.

Participants with anti–Vi-IgG titers above these thresholds (seropositive) were followed up on day 180, and 2 stool specimens were collected 48 hours apart to detect *S*. Typhi. Further follow-up was performed of participants identified as possible asymptomatic carriers (defined as seropositive participants with stool culture positive for *S*. Typhi).

### Laboratory Procedure

Informed written consent was obtained from guardians of young participants (aged 1–17 years), while adult participants (aged 18–59 years) provided their own consent. Stool cultures were performed at the icddr,b laboratory. Inoculation and emulsification of stool in selenite F broth medium for parallel enrichment and direct inoculation to MacConkey and *Salmonella-Shigella* selective agar plates were performed. Incubation of all cultures were carried out at 37°C for 18–24 hours. Colonies of *S*. Typhi were identified by phenotypic morphology, standard biochemical tests, and slide agglutination to *Salmonella*-specific antisera. Antimicrobial susceptibility testing was performed using standard disk diffusion methods [8]. Plasma was separated from participant blood by centrifugation, and anti–Vi-IgG titers were measured using a commercial assay (VaccZyme enzyme-linked immunosorbent assay; The Binding Site), following the manufacturer’s guidelines [9].

### Treatment of Chronic Carriers

Antibiotic treatment was recommended for index patients and their household contacts according to an agreed-upon management plan, if shedding continued for up to 1 year after enrollment. Similarly, antibiotic treatment was also planned for any asymptomatic chronic carriers detected through serosurveillance [7]. After completion of antibiotic therapy, 3 consecutive stool specimens collected ≥1 month apart were required to confirm curative treatment.

### Statistical Analysis

To evaluate the differences among groups, χ2 and Mann-Whitney *U* tests were used ,and results were considered statistically significant at *P*<.05.

## RESULTS

### Passive Surveillance

The total number of individuals included in the study census was 111695, among whom 4509 patients met the inclusion criteria and were enrolled to the passive surveillance study between August 2016 and January 2019. Of these, 332 of 4509 (7%) were confirmed as typhoid cases by blood culture and formed the index patient population and 109 of 332 (33%) were also stool culture positive at enrollment (day 0). In total, stool specimens were collected from 4216 of the 4509 participants at enrollment, and 33 of 4216 (0.8%) were stool culture positive but negative by blood culture.

Among the 332 blood culture–confirmed index patients, stool culture was again positive for 15 of 281 patients (5%) on day 3 and 1 of 270 (0.4%) patients on day 180 (Table 1). None of these patients were found to shed *S*. Typhi 1 year later. In patients with only positive stool cultures, 1 of 9 was positive on day 3, and none were positive at later time points.

**Table 1.** Isolation of *Salmonella* Typhi in Stool Specimens of Blood Culture– and Stool Culture–Positive Patients at Different Time Points

Time After Enrollment, d	BC-Positive Index Patients (n = 332)		SC-Positive Index Patients (n = 33)	
	Stool Specimen Collected for SC, No.	SC Positive for *S*. Typhi, No. (%)	Stool Specimen Collected for SC, No.	SC Positive for *S*. Typhi, No. (%)
30	281	15 (5)	9	1 (11)
180	270	1 (0.4)	9	0 (0)
365^a^	15	0 (0)	1	0 (0)

Abbreviations: BC, blood culture; SC, stool culture.

a Stool specimens were collected on day 365 if SCs were positive for *Salmonella* Typhi on day 30 and/or day 180.

For household contacts of blood culture–confirmed index patients (n=332), 530 household contacts from 217 households (2.4 contacts per household) were enrolled in the study. Stool specimens were obtained from 530 and 466 household contacts on days 0 and 30 and tested for culture. The median age of household contacts was 29 years (range, 5 months to 75 years), and the male-female ratio of the contacts was 1:1.5. Among them, 5 of 530 participants (0.9%) and 4 of 466 (0.7%) were *S*. Typhi positive in stool on days 0 and 30, respectively (Table 2). No shedding was revealed on day 180 among 7 contacts from whom stool specimens were obtained, and no stool was therefore collected at 1 year.

**Table 2.** Isolation of *Salmonella* Typhi in Stool Specimens From Household Contacts of Blood Culture– and Stool Culture–Positive Patients at Different Time Points

Time After Enrollment, d	HH Contacts of BC-Positive Index Patients		HH Contacts of SC-Positive Index Patients	
	Stool Specimen Collected for SC, No.	SC Positive for *S*. Typhi, No. (%)	Stool Specimen Collected for SC, No.	SC Positive for *S*. Typhi, No. (%)
0	530	5 (0.9)	9	0 (0)
30	466	4 (0.7)	9	0 (0)
180^a^	7	0 (0)	0	0 (0)

Abbreviations: BC, blood culture; HH, household; SC, stool culture.

a Stool specimens were collected on day 180 if SCs were positive for *Salmonella* Typhi on day 0 and/or day 30.

Of 332 blood culture–confirmed index patients, 16 from 8 households (2 patients per household) had typhoid fever episodes at the same time within their households. Among them, 1 pair of index patients of the same household were also shedding the bacterium at the same time.

For index patients who were stool culture positive only (n=33), 9 household contacts from 5 households (1.8 contacts per household) were enrolled in the study. The median age of the contacts was 29 years (range, 8–48 years), and the male-female ratio was 1:2. Stool specimens were collected from them and tested for culture on days 0 and 30, and none were found to shed *S*. Typhi at these time points (Table 2).

Exploratory analyses of baseline characteristics (age, temperature, and duration of fever) demonstrated no significant differences between patients who were positive by both blood and stool culture and those who were positive only by blood culture. However, the proportion of prior antibiotic usage for the blood culture– and stool culture–positive patients (24 of 109 [22%]) was significantly lower (*P*≤.05) than that in patients who were only blood culture positive (90 of 223 [40%]).

### Serosurvey Component

Paired blood specimens were collected from 8261 and 7043 participants on days 0 and 90 respectively. High anti–Vi-IgG titers were measured in 303 participants on day 0 and/or 90, among whom 136 had persistently high Vi for both time points. Two stool specimens 48 hours apart were collected from 192 of 303 participants on day 180.

Only 1 woman aged 36 years was *S*. Typhi positive by stool culture and therefore identified as an asymptomatic chronic (probable) carrier and treated with ciprofloxacin (750mg twice daily for 28 days). No *S*. Typhi shedding was detected in 3 consecutive stool specimens collected after completion of antibiotic treatment. Ultrasonography of the hepatobiliary system revealed gallstones, and further advice was provided regarding consideration for elective cholecystectomy.

## DISCUSSION

The study results demonstrate the high proportion of patients who shed *S*. Typhi in their stool during the acute stages of typhoid fever. This is a potential high-risk period for human-to-human transmission to occur, given the likely debilitation of the patient and their care needs. Those at greatest risk would include household members and caregivers. In contrast, very low levels of continued shedding were seen during treatment and convalescence stages, likely a reflection of high levels of effectiveness of modern antimicrobial therapy. No study participants had continued shedding into the chronic carriage stage. Although no difference was found in the duration of fever between patients who were blood and stool culture positive and those who were only blood culture positive, but an association of early *S*. Typhi shedding and development of typhoid fever has also been described in the human challenge model study performed in Oxford, United Kingdom [10]. History of prior antibiotic intake was observed in more blood culture–positive patients who were also shedding *S.* Typhi than in those who were only blood culture positive. Similarly, after initiation of antibiotic in human challenge model, no shedding was seen [10].

Participants who were stool culture positive but blood culture negative, indicating either recent infection with short-term shedding or a false-negative blood culture, responsible for transmitting the organism in the community. Hence, they were followed up in the same way of blood culture–positive index patients and household contacts being screened. However, stool specimen collection and retesting on day 30 was possible for 9 of 33 stool culture–positive index patients (27%), 1 of whom was positive for *S*. Typhi. Therefore, it is difficult to make an inference from this study result regarding the rates and duration of bacterial shedding and the impact of patients who are only stool culture positive in spreading the organism.

The household contact study revealed that some household members of the acute patients shed *S.* Typhi in the absence of typhoid fever symptoms and during the active disease period of the index patients. In addition, some blood culture–positive index patients had typhoid fever episode within a household at the same time, among whom 2 index patients also had bacterial shedding in their stool. In support of our findings, many epidemiological studies have reported that *S*. Typhi is transmitted from convalescent carriers to close contacts of patients within their household [11, 12].

One person was confirmed as an asymptomatic carrier identified by serosurveillance. Increased age, female sex, and gallbladder disease are risk factors for chronic carriage after recovery from acute typhoid disease [3] and were present in the individual identified.

There are several important points to consider in interpreting the low rates of carriage found in this study. First, the chronic carriage prevalence may be low, and asymptomatic carriers may not make any major contribution to ongoing transmission in the community. Second, *S*. Typhi are excreted in feces in low numbers and intermittently, which makes the detection of carriers difficult, and thus true prevalence may have been missed. Third, anti–Vi-IgG may not be the most appropriate biomarker for serological identification of chronic carriers in endemic settings. However, measurement of anti–Vi-IgG is widely considered a potentially useful approach for identifying chronic carriers, since increased levels of anti–Vi-IgG are highly prevalent in apparently healthy individuals in typhoid-endemic areas [13]. Therefore, we decided to use this biomarker to increase the probability of detecting stool shedding in asymptomatic carriers in this setting. Its use is also recommended for investigating the outbreaks in nonendemic settings [14].

The study findings highlight that (1) patients with acute typhoid have the highest risk of shedding and therefore transmission (even in presymptomatic stages), (2) the use of anti–Vi-IgG is not helpful, and (3) using resources to find cases within households has a low success rate and so should be considered carefully in setting priorities.

In conclusion, accurate diagnosis and appropriate treatment are urgently needed in patients with acute typhoid as well as in short-term convalescent carriers to prevent outbreaks and eliminate typhoid fever from endemic countries. Similarly, better approaches are needed for surveillance and population-level detection of carriers in endemic settings, as vaccine is rolled out and carriage becomes more of an issue and barrier to eradication [5].

## Notes

***Acknowledgments.*** We acknowledge the support of the study participants as well as the dedicated field and laboratory workers in this study at icddr,b, and icddr,b is grateful to the governments of Bangladesh, Canada, Sweden, and the United Kingdom for providing core/unrestricted support.

***Financial support.*** This work was supported by the Bill & Melinda Gates Foundation (grant 617 OPP1141321) and the Wellcome Trust (Strategic Award no. 106158/Z/14/Z).

***Potential conflicts of interest.*** All authors: No reported conflicts. All authors have submitted the ICMJE Form for Disclosure of Potential Conflicts of Interest. Conflicts that the editors consider relevant to the content of the manuscript have been disclosed.

## References

[1] Mitscherlich E, Marth EH. Microbial survival in the environment: bacteria and rickettsiae important in human and animal health. Berlin and New York: Springer-Verlag; 1984. p. ix, 802.

[2] Gonzalez-Escobedo G, Marshall JM, Gunn JS. Chronic and acute infection of the gall bladder by *Salmonella* Typhi: understanding the carrier state. Nat Rev Microbiol 2011; 9:9–14. 21113180 10.1038/nrmicro2490 PMC3255095

[3] Levine MM, Black RE, Lanata C. Precise estimation of the numbers of chronic carriers of *Salmonella* typhi in Santiago, Chile, an endemic area. J Infect Dis 1982; 146:724–6. 7142746 10.1093/infdis/146.6.724

[4] Parry CM, Hein TT, Dougan G, et al. Typhoid fever. N Engl J Med 2002; 347:1770–82. 12456854 10.1056/NEJMra020201

[5] Gunn JS, Marshall JM, Baker S, Dongol S, Charles RC, Ryan ET. *Salmonella* chronic carriage: epidemiology, diagnosis, and gallbladder persistence. Trends Microbiol 2014; 22:648–55. 25065707 10.1016/j.tim.2014.06.007 PMC4252485

[6] Bokkenheuser V. Detection of typhoid carriers. Am J Public Health Nations Health 1964; 54:477–86. 14128664 10.2105/ajph.54.3.477 PMC1254745

[7] Darton TC, Meiring JE, Tonks S, et al; STRATAA Study Consortium. The STRATAA study protocol: a programme to assess the burden of enteric fever in Bangladesh, Malawi and Nepal using prospective population census, passive surveillance, serological studies and healthcare utilisation surveys. BMJ Open 2017; 7:e016283. 10.1136/bmjopen-2017-016283 PMC5726077 28674145

[8] Wayne P. Clinical and laboratory standards institute. Performance standards for antimicrobial susceptibility testing. Informational Supplement. CLSI. 2011; 31:100–21.

[9] Mohan VK, Varanasi V, Singh A, et al. Safety and immunogenicity of a Vi polysaccharide-tetanus toxoid conjugate vaccine (Typbar-TCV) in healthy infants, children, and adults in typhoid endemic areas: a multicenter, 2-cohort, open-label, double-blind, randomized controlled phase 3 study. Clin Infect Dis 2015; 61:393–402. 25870324 10.1093/cid/civ295

[10] Waddington CS, Darton TC, Jones C, et al. An outpatient, ambulant-design, controlled human infection model using escalating doses of *Salmonella* Typhi challenge delivered in sodium bicarbonate solution. Clin Infect Dis 2014; 58:1230–40. 24519873 10.1093/cid/ciu078 PMC3982839

[11] Karkey A, Thompson CN, Tran Vu Thieu N, et al. Differential epidemiology of *Salmonella* Typhi and Paratyphi A in Kathmandu, Nepal: a matched case control investigation in a highly endemic enteric fever setting. PLoS Negl Trop Dis 2013; 7:e2391. 23991240 10.1371/journal.pntd.0002391 PMC3749961

[12] Dongol S, Thompson CN, Clare S, et al. The microbiological and clinical characteristics of invasive *Salmonella* in gallbladders from cholecystectomy patients in Kathmandu, Nepal. PLoS One 2012; 7:e47342. 23077595 10.1371/journal.pone.0047342 PMC3471863

[13] Gupta A, My Thanh NT, Olsen SJ, et al. Evaluation of community-based serologic screening for identification of chronic *Salmonella* typhi carriers in Vietnam. Int J Infect Dis 2006; 10:309–14. 16412678 10.1016/j.ijid.2005.06.005

[14] Olsen SJ, Bleasdale SC, Magnano AR, et al. Outbreaks of typhoid fever in the United States, 1960-99. Epidemiol Infect 2003; 130:13–21. 12613741 10.1017/s0950268802007598 PMC2869934

